# MicroRNA Transcriptome Profiling in Heart of *Trypanosoma cruzi*-Infected Mice: Parasitological and Cardiological Outcomes

**DOI:** 10.1371/journal.pntd.0003828

**Published:** 2015-06-18

**Authors:** Isabela Cunha Navarro, Frederico Moraes Ferreira, Helder I. Nakaya, Monique Andrade Baron, Gláucia Vilar-Pereira, Isabela Resende Pereira, Ana Maria Gonçalves Silva, Juliana Monte Real, Thales De Brito, Christophe Chevillard, Joseli Lannes-Vieira, Jorge Kalil, Edecio Cunha-Neto, Ludmila Rodrigues Pinto Ferreira

**Affiliations:** 1 Laboratory of Immunology, Heart Institute (InCor), University of São Paulo School of Medicine, São Paulo, Brazil; 2 Division of Clinical Immunology and Allergy, University of São Paulo School of Medicine, São Paulo, Brazil; 3 Institute for Investigation in Immunology, iii-INCT, São Paulo, Brazil; 4 Department of Clinical Analyses and Toxicology, School of Pharmaceutical Sciences, University of São Paulo, São Paulo, Brazil; 5 Laboratory of Biology of Interactions, Oswaldo Cruz Institute—FIOCRUZ, Rio de Janeiro, Brazil; 6 Institute of Tropical Medicine, University of São Paulo, São Paulo, Brazil; 7 Department of Pathology, University of São Paulo School of Medicine, São Paulo, Brazil; 8 Hospital Sírio-Libanês, São Paulo, Brazil; 9 INSERM, U906, Aix-Marseille University AMU, Faculté de Médecine, Marseille, France; Albert Einstein College of Medicine, UNITED STATES

## Abstract

Chagas disease is caused by the parasite *Trypanosoma cruzi*, and it begins with a short acute phase characterized by high parasitemia followed by a life-long chronic phase with scarce parasitism. Cardiac involvement is the most prominent manifestation, as 30% of infected subjects will develop abnormal ventricular repolarization with myocarditis, fibrosis and cardiomyocyte hypertrophy by undefined mechanisms. Nevertheless, follow-up studies in chagasic patients, as well as studies with murine models, suggest that the intensity of clinical symptoms and pathophysiological events that occur during the acute phase of disease are associated with the severity of cardiac disease observed during the chronic phase. In the present study we investigated the role of microRNAs (miRNAs) in the disease progression in response to *T*. *cruzi* infection, as alterations in miRNA levels are known to be associated with many cardiovascular disorders. We screened 641 rodent miRNAs in heart samples of mice during an acute infection with the Colombiana *T*.*cruzi* strain and identified multiple miRNAs significantly altered upon infection. Seventeen miRNAs were found significantly deregulated in all three analyzed time points post infection. Among these, six miRNAs had their expression correlated with clinical parameters relevant to the disease, such as parasitemia and maximal heart rate-corrected QT (QTc) interval. Computational analyses identified that the gene targets for these six miRNAs were involved in networks and signaling pathways related to increased ventricular depolarization and repolarization times, important factors for QTc interval prolongation. The data presented here will guide further studies about the contribution of microRNAs to Chagas heart disease pathogenesis.

## Author Summary

Chagas’ disease is caused by the protozoan parasite *Trypanosoma cruzi* and affects 8 million individuals worldwide. The life-long infection begins with a short acute phase, which is associated to parasites circulating in the bloodstream, tissue parasitism, and various signs and symptoms including those related to myocarditis. After resolution of the acute phase, about 30% of those chronically infected will develop abnormal ventricular repolarization with hypertrophy, myocarditis and fibrosis by yet undefined mechanisms. MicroRNAs play a key role in silencing gene expression and are essential elements of the physiology and pathophysiology of the cardiovascular system. Here we describe for the first time the effect of acute *T*. *cruzi* infection on host miRNA expression by screening 641 rodent miRNAs in heart samples. A number of miRNAs have significantly altered expression upon infection and several of them correlate with *T*. *cruzi* parasitism and electrocardiographic changes. Pathway analysis results suggest that these dysregulated miRNAs can potentially affect gene networks and signaling pathways related to increased ventricular depolarization and repolarization times. Our study provides new insights on miRNA regulation of genes relevant to parasitological and cardiological outcomes.

## Introduction

Chagas disease is caused by the infection of an intracellular protozoan parasite, *Trypanosoma cruzi*, and affects 8 million individuals worldwide [1]. The acute phase of the disease is associated to parasites circulating in the bloodstream and intense tissue parasitism [2]. Patients with severe symptoms of acute Chagas disease present myocarditis, with myocardial dyskinesis, heart enlargement and heart failure [3]. Alterations in echocardiography (ECHO) and electrocardiography (ECG) such as disturbances of ventricular repolarization, pericardial effusion and atrial fibrillation during the acute phase of Chagas disease are associated with poor prognosis [4]. After resolution of the acute phase, chronically infected patients display sub patent parasitemia with low tissue parasitism and an asymptomatic (indeterminate) form of the disease. The indeterminate form of chronic Chagas disease may evolve to a cardiac form in 30% of patients called chronic chagasic cardiomyopathy (CCC), which is characterized by dilatation of the heart, fibrosis, arrhythmias, heart conduction abnormalities and sudden death [5]. The study of Chagas´ heart disease has been aided by the use of murine model of *T*. *cruzi* infection, which recapitulates many of the functional and pathological alterations of the human disease, including myocarditis, observed during the acute phase of infection with this parasite [6]. There is evidence that the intensity of symptoms in the acute phase may be positively correlated to the severity of the cardiac disease in the chronic phase [7,8].

MicroRNAs (miRNAs) represent a class of small non-coding RNAs (typically 19–23 nucleotides) [9] which act by annealing to partially complementary binding sites present on the 3' untranslated regions (UTR) of messenger RNAs (mRNAs) leading to inhibition of protein translation or by inducing mRNA decay. In mammals, miRNA regulate tissue-specific protein expression and are involved in virtually all cellular processes. Up to one-third of mammalian mRNAs are susceptible to miRNA-mediated regulation [10]. It has been shown that miRNAs are determinants of the physiology and pathophysiology of the cardiovascular system and altered expression of muscle- and/or cardiac-specific miRNAs such as the miRNAs named miR-1, miR-208 and miR-133 in myocardial tissue is involved in heart development and cardiovascular diseases, including myocardial hypertrophy, heart failure and fibrosis [11–14]. Previous work from our group has shown for the first time that the miRNA expression is dysregulated in CCC. In the study, we have found that the same muscle- and/or cardiac-specific miRNAs, miR-1, miR-133 and miR-208 were downregulated in CCC myocardium as compared to control myocardium [15].

However, no study has approached the expression of miRNAs during the acute phase of Chagas disease. To investigate the consequences of acute *T*. *cruzi* infection in host miRNA expression, we used TaqMan Low Density Arrays (TLDA) to screen 641 miRNAs in mouse heart samples at 15, 30 and 45 days post *T*. *cruzi* infection (dpi). The results revealed that a large number of miRNAs have significantly altered expression upon infection. In addition, the number of differentially expressed miRNAs among the three *T*. *cruzi*-infected groups increased over time. We have identified a cluster of 17 miRNAs significantly deregulated in all three different time points examined post infection. Several associated with clinical features, such as blood parasitemia and prolongation of ventricular depolarization and repolarization time or QTc interval. Pathway analysis revealed that the gene targets of six miRNAs, which were differentially expressed during all three time points post infection and are also associated with both clinical parameters, belong to signaling pathways related to QTc interval and parasitemia.

## Materials and Methods

### Ethics statement

This study was carried out in strict accordance with the recommendations in the Guide for the Care and Use of Laboratory Animals of the Brazilian National Council of Animal Experimentation (http://www.cobea.org.br/) and the Federal Law 11.794 (October 8, 2008). The Institutional Committee for Animal Ethics of Fiocruz (CEUA/Fiocruz, License 004/09) and the Institutional Committee for Animal Ethics of the University of São Paulo School of Medicine (CEUA/FMUSP, License 390/13) approved all the procedures used in this study.

### Mouse and parasite strains

A total of 48 pathogen-free C57BL6 female mice (6- to 7-wk-old) from the FIOCRUZ animal facility (CECAL, Rio de Janeiro, Brazil) were used in the current study. Colombiana *T*. *cruzi* strain was continuously maintained in C57BL/6 mice (weight, 18 to 20g).

### Experimental infections, parasitemia and mortality assessment

Thirty-six C57BL6 mice (females; weight, 20 to 25g), were intraperitoneally infected with 100 blood Colombiana *T*. *cruzi* Type I strain trypomastigotes for 15, 30 and 45 days (12 mice per time point). The level of parasitemia in tail blood was assessed daily by the Brener' s method [16].

### Histologic evaluation of *T*. *cruzi*-infected mice

Hearts were fixed in buffered formalin solution, embedded in paraffin, and cut into 5 μm sections. Sections were stained with haematoxylin-eosin or used for the detection of *T*. *cruzi* antigens by means of the immunoperoxidase method. For the latter use, sections were deparaffinised and incubated with polyclonal anti-*T*. *cruzi* antiserum obtained from rabbits immunized with *T*. *cruzi* whole homogenate. Biotinylated goat anti–mouse/rabbit IgG and Duet Strepto ABComplex (Dako, Denmark) were used as a secondary antibody and for amplification, respectively. Diaminobenzadine (SIGMA Chemical Co, USA) was used as chromogen. preimmune rabbit serum was used as a negative control [17].

### Electrocardiograms (ECG)

Mice were tranquilized with diazepam (10mg/kg) and transducers were placed subcutaneously (DII). The traces were recorded for 2 min using a digital Power Lab 2/20 system connected to a bio-amplifier at 2 mV for 1 second (PanLab Instruments, Spain). The filters were standardized to between 0.1 and 100 Hz and the traces were analyzed using Scope software for Windows V3.6.10 (PanLab Instruments, Spain). The ECG parameters were analyzed as previously described [18].

### Isolation of total RNA

After each time point (15, 30 and 45 dpi), ventricles isolated from each heart were mechanically disrupted with the Precellys 24-bead-based homogenizer (Bertin Technologies, France) using 3 cycles of 15 seconds at 6,000 rpm, between intervals of 20 seconds. Total RNA was isolated from samples homogenized in 500μl of lysis buffer from mirVana miRNA Isolation Kit (Ambion, USA), following the manufacturers’ protocol. RNA concentration and purity was measured using a NanoDrop-1000 spectrophotometer (Thermo Scientific, USA) and integrity was determined on a Bioanalyzer 2100 (Agilent, USA). Only samples with RIN (RNA Integrity Number) value > 8.0 were used in our analyses.

### Quantitative miRNA expression profiling

Four successfully infected mice per group (15, 30 and 45 dpi) were used for heart expression profiling experiment of 641 miRNAs according to the Applied Biosystems protocols. Briefly, reverse transcription was performed with 500ng total RNA using Megaplex RT stem loop primers, Multiscribe Reverse Transcriptase, RNase inhibitor and 100nM deoxynucleotide triphosphates (dNTPs) (reagents from Applied Biosystems, Life Technologies, USA). The multiplexed RT reaction was performed according to manufacturer’s instructions. Quantitative real-time RT-PCR was done utilizing pre-printed TLDA microfluidic cards (Rodent Card A + B v3, format 384 each). Each card set contained MGB labelled probes specific to mature miRNAs plus endogenous small nucleolar RNAs for data normalization and relative quantification. The sample/master mix for each Megaplex pool was loaded into the cards, centrifuged and mechanically sealed with the Applied Biosystems sealer device. Real time-PCR reaction was carried out on an Applied Biosystems 7900HT thermocycler using the cycling conditions: 40 cycles of 2 minutes at 16°C, 1 minute at 42°C, 1 second at 50°C and 5 minutes at 85°C. Raw TLDA data files were pre-processed with threshold and baseline corrections for each sample (automatic baseline and threshold set to 0.3) with each amplification plot assessed to confirm that the threshold cycle (Ct) value corresponded with the midpoint of logarithmic amplification (SDS 2.3, Life Technologies, USA).

### Statistical analysis

MicroRNA statistical analysis was carried out using the Linear Model for microarray data (Limma) with adjustment for false discovery rate with the Benjamini-Hochberg [19,20] method using RealTime StatMiner software (Integromics, Version 4.0). The comparative threshold cycle method was used to calculate the relative miRNA expression after global normalization [21]. Statistical significance threshold was defined as p≤ 0.05. The hierarchical clustering was performed using squared Euclidean as distance measure and Ward's method for linkage analysis and z-score normalization. The principal component analysis (PCA) plot of samples was performed using all probe sets by using the median centering of the data set. Correlation analyses were performed based on Peason correlation with GraphPad Prism software (version 5.0.4). Unbiased Pearson correlation analyses were performed by crossing the Ct values of all the 641 microRNAs assayed over parasitemia and QTc interval values, using R software (R Development Core Team, Austria– www.R-project.org). For miRNA and mRNA expression analysis by real time PCR, groups were compared by a non-parametrical test (*Mann-Whitney Rank Sum Test*) with GraphPad Prism software (version 5.0.4). Results were expressed as medians and interquartile ranges. *P*-values were considered significant if <0.05 (marked with a * symbol).

### Target prediction and network pathway analysis

The software Ingenuity Pathways Analysis (IPA) (Qiagen, USA- www.ingenuity.com) and the tool called “target filter” which relies on three popular algorithms (TargetScan, TarBase and miRecords) was used to identify putative targets of the six miRNAs differentially expressed in the heart of infected mice. These six miRNAs were selected because they were differentially expressed in all three time-points post infection and had the highest correlation significance with both clinical parameters: parasitemia and QTc interval parameters. IPA Network maintains a graphical database of networks of interacting genes (Ingenuity Knowledge Base, IKB). A list containing the six miRNAs was uploaded in the IPA and analyzed based on the content of date 2014–07. The significance of the association between each list and the pathway was measured by Fisher’s exact test. As a result, a *P*-value was obtained, determining the probability that the association between the genes in our data set and the network generated can be explained by chance alone. Molecules are represented as nodes, and the biological relationship between two nodes is represented as an edge (line). All edges are supported by at least one reference from the literature, from a textbook, or from canonical information stored in the IKB.

### Analysis of miRNA and mRNA expression by real-time RT-PCR

Real-time RT-PCR reactions were performed using TaqMan microRNA and mRNA expression assays (Applied Biosystems, Foster City, CA). RT reactions of the mature microRNAs: miR149-5p [assay ID 002255], miR-21-5p [assay ID 000397], miR145-5p [assay ID 002278] and miR142-5p [assay ID 002248] were performed using 100 ng of enriched miRNA total RNA, 50 n*M* stem-loop RT primer, 10× RT buffer, 100 mM each dNTPs, 50 units/μl of MultiScribe reverse transcriptase, and 20 units/μl of RNase inhibitor. Reaction mixtures (15 μl) were incubated in the thermocycler (Applied Biosystems) for 30 minutes at 16°C, 30 minutes at 42°C, and 5 minutes at 85°. Real-time PCR was carried out in a 10 μl PCR mixture containing 1.33 μl of RT product, 2× TaqMan Universal PCR Master Mix, 0.2 μM TaqMan probe, 15 μM forward primer, and 0.7 μM reverse primer. For the genes expression analysis (CACAN1C [assay ID Mm01188822_m1*], KCNA1 [assay ID Mm00439977_s1], SLC18A2 [assay ID Mm00553058_m1*], GJA5 [assay ID Mm00433619_s1*], and RNF207 [assay ID Mm01260273_m1*]), a total of 3 μg RNA were reverse transcribed using oligo-dT and a set of random primers (Invitrogen). Real-time quantitative RT-PCR was executed in a 20μL reaction containing the cDNA PCR Master Mix (Applied Biosystems) and the specific Taq-Man primers/probe. The 18S mRNA expression was used for normalization. The reactions were performed in the QuantStudio12K PCR System (Applied Biosystems). Sets of “no RT” controls were included for each experiment. Reactions were performed in triplicate and the threshold cycle (Ct) values averaged for replicates. Expression was calculated as median ± interquartile ranges per group for each individual data point using the 2^-ΔΔCt^ relative expression equation (fold change over control samples) [22].

## Results

### Parasitemia, survival and prolongation of QTc interval in acutely *T*. *cruzi* infected mice

**Fig 1:**
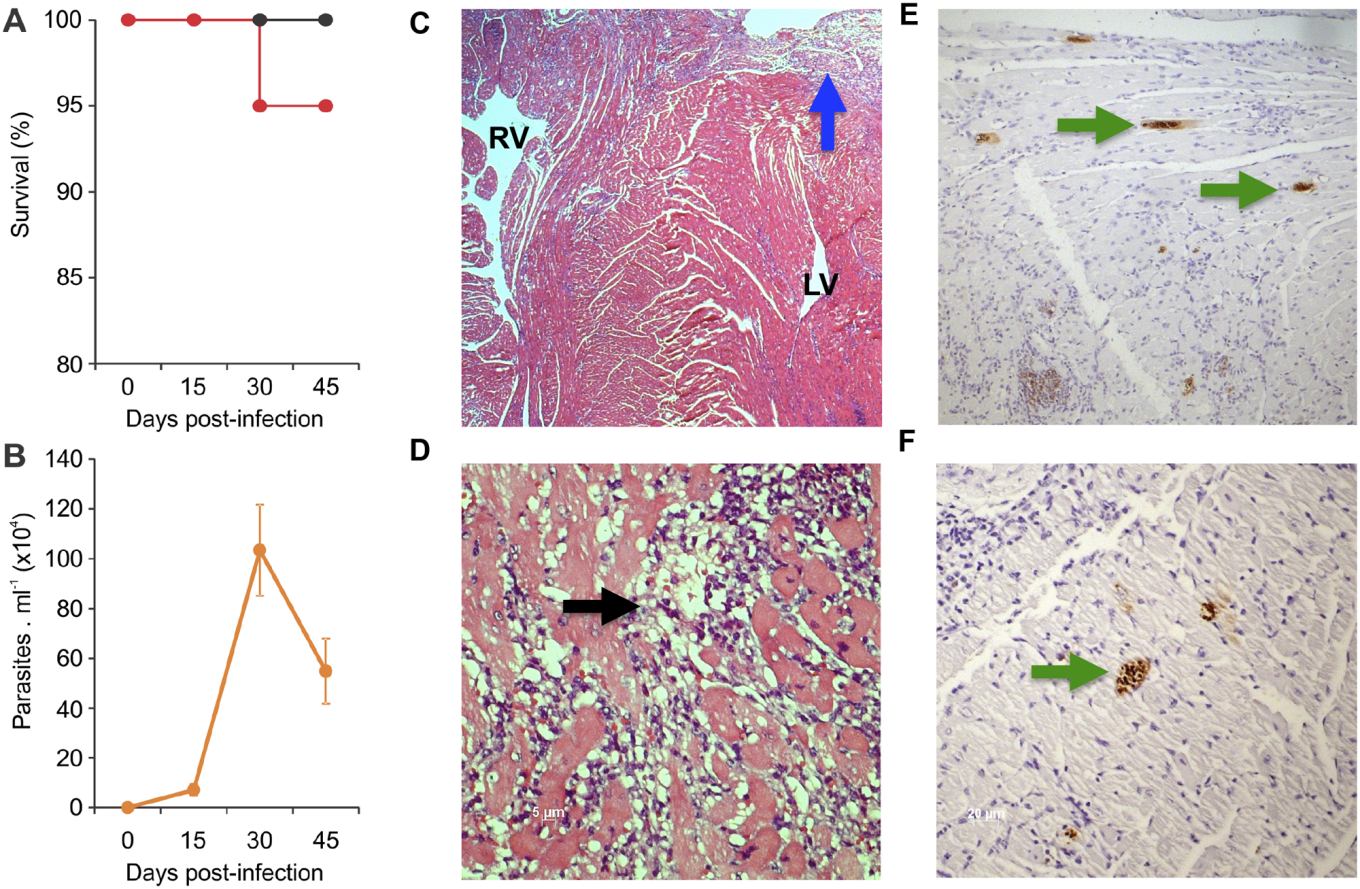
(A) Survival rate, (B) parasitemia and (C to F) histological evaluation of cardiac tissue. A-B: Mice were infected with 100 blood trypomastigotes of the Colombian strain of *T*. *cruzi* and the (A) survival rate and (B) parasite load were evaluated 15, 30 or 45 days after infection. Each point represents the group mean and the vertical bars represent the standard error (n = 12 animals per group). C-F: Representative images of histological characteristics of infected animals after (C and D) haematoxylin and (E and F) eosin or anti-*T*. *cruzi* staining. The arrows indicate (C; inflammatory infiltrate; blue arrow; 45 dpi; 2,5x), myocytes vacuolization, myocarditis and fiber necrosis (D; 45 dpi; black arrow; 40x) and amastigote nests (E and F; green arrows; 45 dpi; 10x and 20x).

**Fig 2:**
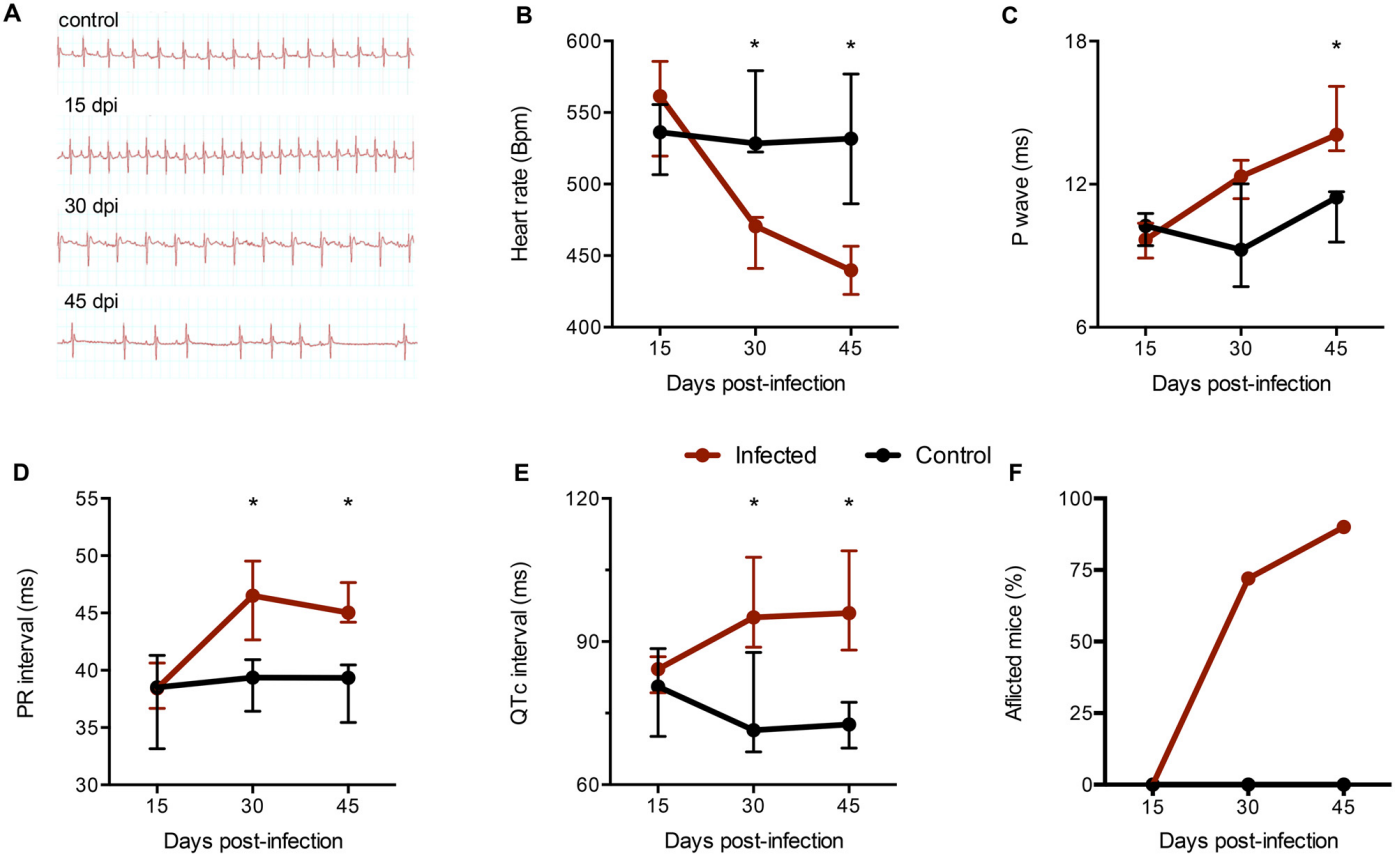
Electrocardiographic alterations in infected animals. Mice were tranquilized with Diazepam (10 mg/Kg) and the transducers were carefully placed subcutaneously according to chosen preferential derivation (DII). The traces were recorded for 2 minutes. A: Representative ECG registers segments showing second degree atrioventricular block (30 dpi) and arrhythmia (45 dpi). B-E: Electrocardiographic parameters evaluated 13, 30 or 45 dpi. Results were expressed as medians and interquartile ranges (n = 12 animals per group). Groups were compared by a non-parametrical test (Mann-Whitney Rank Sum Test) with GraphPad Prism software (version 5.0.4). * p < 0,05. F: Percentage of afflicted animals at each time point.

C57BL/6 mice were intraperitoneally infected with 100 blood trypomastigotes of Colombian *T*. *cruzi* strain. This parasite strain was previously demonstrated to have a tissue tropism to skeletal muscle and myocardium and high pathogenicity [23]. Survival and parasitemia were assessed at 15, 30 and 45 dpi (12 mice per group). As shown in Fig 1A and 1B, the onset of the acute phase was associated with only 5% of mortality and an increase in parasitemia at 15 dpi, with an peak at 30 dpi (as expected for the infection with Colombian strain with the inoculum used [18]). Fig 1C–1F present representative images of histological characteristics of infected animals after haematoxylin and eosin (Fig 1C and 1D) or anti-*T*. *cruzi* staining (Fig 1E and 1F). Fig 1C shows an intense infiltrate of inflammatory cells in both ventricles of the myocardium (at 45 dpi), while Fig 1D shows in higher magnification the myocarditis, myocytes vacuolization, and necrotizing fibers (at 45 dpi). Fig 1E and 1F show the presence of amastigote nests at 45 dpi at 10X and 20X magnification, respectively. Fig 2A presents the ECG profiles of the uninfected controls and all three time points post infection showing the second-degree atrioventricular block and arrhythmia at 30 dpi and 45 dpi. Infection with *T*. *cruzi* induced the first ECG alterations at 30 dpi with significant alterations in heart rate (Fig 2B), prolongation of P wave (Fig 2C) and PR interval (Fig 2D). Fig 2E shows QTc interval prolongation starting at 30 dpi. And finally, Fig 2F shows that 75% and 90% of the infected mice presented ECG alterations, at 30 and 45 dpi, respectively.

### *T*. *cruzi* infection significantly alter the expression of miRNAs

**Fig 3:**
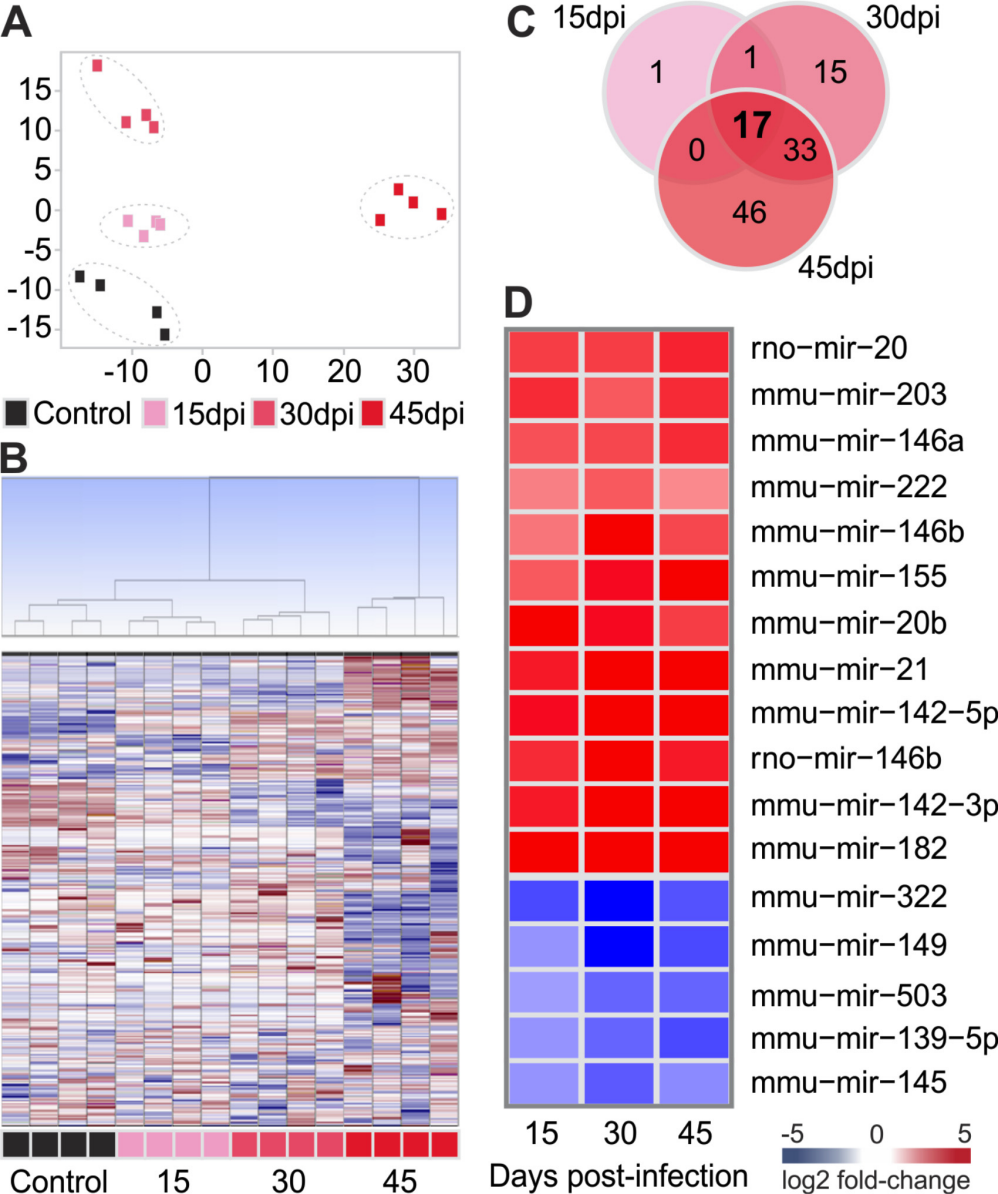
Characterization of microRNA expression profile. A: Principal component analysis (PCA) plot of samples was performed using all probe sets, by using a median centering of the data set. B: Hierarchical clustering based on squared Euclidean distance measure and Ward's method for linkage analysis and Z score normalization. Each line represents one microRNA and each column represents one sample. The colour scale illustrates the microRNAs relative expression (ΔCt) after global normalization; red and blue represents upregulation and downregulation, respectively. Each square represents one sample. C: Venn diagram showing the number of differentially expressed microRNAs in each time point. D: Heat-map showing the fold change over control for the 17 miRNAs expressed in all time points post infection. The colour scale illustrates the fold change in microRNAs expression relative to uninfected controls; red and blue represents upregulation and downregulation respectively. Each square represents the group mean.

TLDA was used to screen 641 rodent miRNAs in heart samples from acutely *T*. *cruzi* infected mice at 15, 30 and 45 dpi (four mice per group). Principal component analysis (PCA) (Fig 3A) and unsupervised hierarchical clustering (Fig 3B) were performed for all samples and all miRNA assays. Based on their expression values, samples from each time group (Control, 15, 30 and 45 dpi) clustered together, thus confirming homogeneity of the miRNA expression profiles within each group. The group of infected samples clustered independently from the control group. We have found 113 out of 641 miRNAs with significantly altered expression upon infection in at least one time point (S1 Table—in bold). Notably, the number of differentially expressed microRNAs among the three *T*. *cruzi*-infected groups increased over time (Fig 3C). Nineteen microRNAs exhibited differential expression on day 15 post infection. Such numbers increased to 66 and 96, on days 30 and 45, respectively. Seventeen miRNAs were significantly deregulated in all three time points post infection (Fig 3C). Also, 90% of the prematurely altered microRNAs (15 dpi) kept differential expression in all three analysed time points post infection (Fig 3D).

### miRNA expression correlates with clinical parameters: Parasitemia and QTc interval

**Fig 4:**
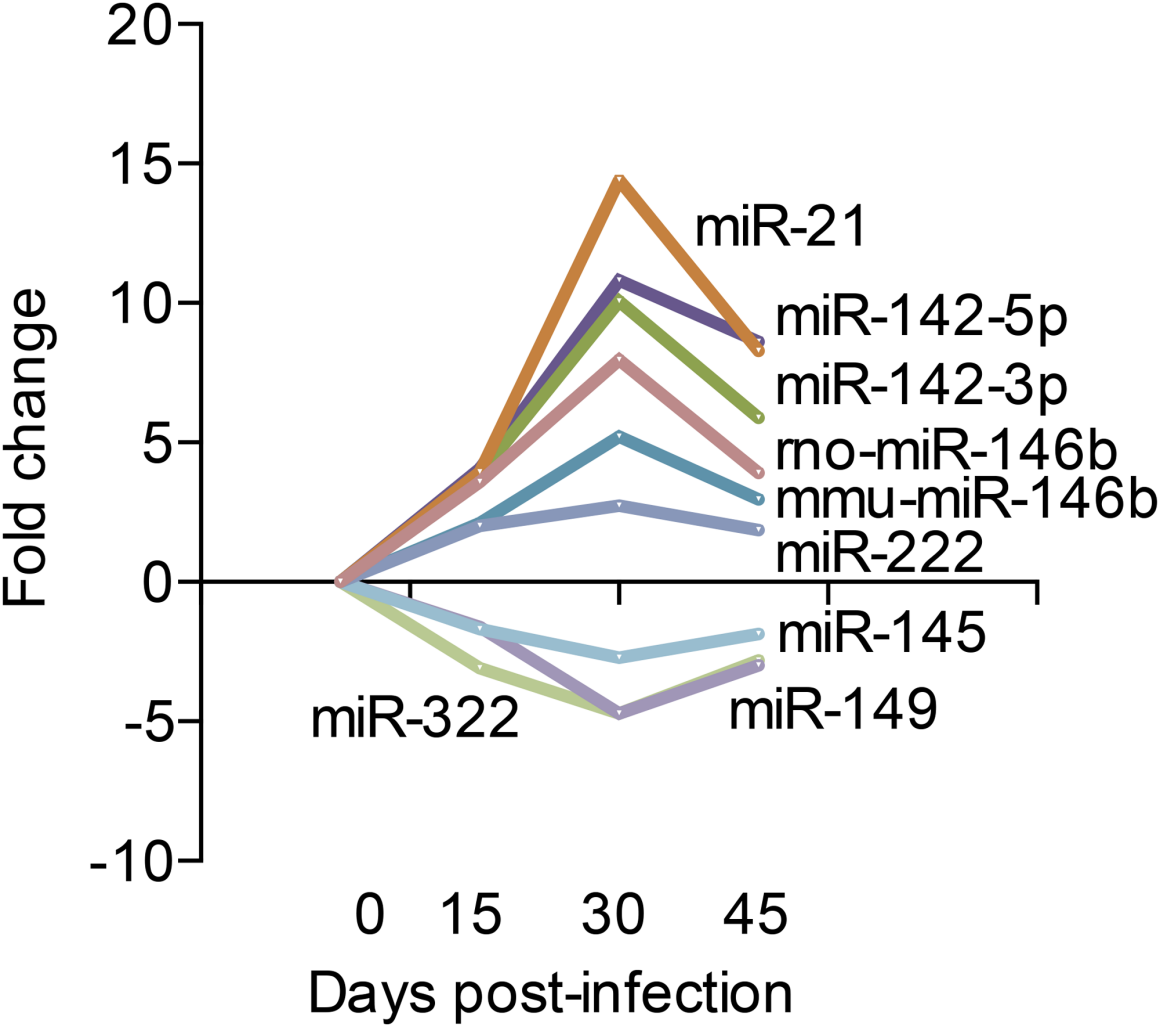
Kinetics of microRNA expression during infection. The line represents the fold change in microRNAs expression in each time point (15, 30 and 45 dpi) relative to uninfected controls (0 dpi).

**Fig 5:**
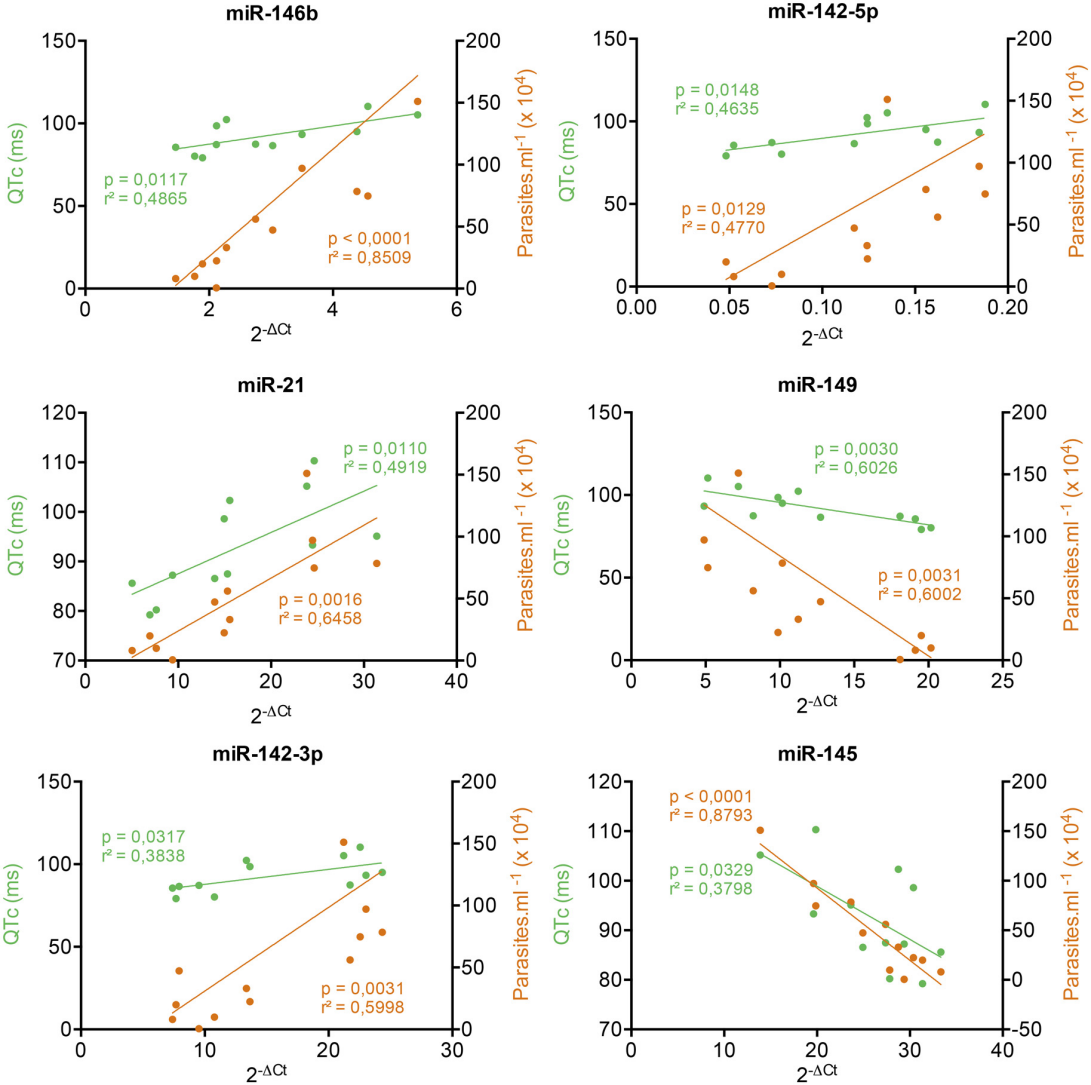
Correlations between microRNA expression and parasitemia and QTc interval alterations. Each point represents one sample; green and orange represents correlation with QTc interval and parasitemia respectively. Lines represent the linear regression for Pearson correlation, p < 0, 05.

Fig 4 shows the expression kinetics of nine miRNAs out of those 17 expressed in all three time points post infection. It is observed that the expression follows a similar profile to that of the parasitemia with a peak at 30 dpi (Fig 1B). These findings suggest that this cluster of nine microRNAs might be, in some way, associated to the magnitude of the infection and indirectly to cardiac alterations. In addition, six (out of nine) microRNAs were significantly correlated with changes in both parasitemia and QTc interval: miR-146b, miR-21, miR-142-3p miR-142-5p (positive correlation) and miR-145-5p and miR-149-5p (negative correlation) (Fig 5). In order to expand and confirm these findings, Pearson correlation analysis was performed crossing the data of all microRNAs—irrespective of whether their expression was significantly different from controls—with the two clinical parameters evaluated, parasitemia and QTc interval (S2 and S3 Tables). It was observed a significant correlation for additional 73 microRNAs with parasitemia (S2 Table), 67 with QTc interval (S3 Table) and 16 with both parameters (Table 1).

**Table 1:** Correlations between microRNA expression and both parameters: Parasitemia and QTc interval.

MicroRNA	R	p-value
mmu-miR-376a	0.874	0.000201
**mmu-miR-146b**	**0.872**	**0.000216**
mmu-miR-696	-0.845	0.000547
mmu-miR-29a	0.839	0.000649
**mmu-miR-149-5p**	**-0.819**	**0.00111**
**mmu-miR-21**	**0.814**	**0.00128**
mmu-miR-342	0.779	0.00281
mmu-miR-34b	0.777	0.00296
mmu-miR-542	-0.777	0.00297
mmu-miR-15b	0.776	0.00298
**mmu-miR-142-3p**	**0.773**	**0.00321**
**mmu-miR-142-5p**	**0.767**	**0.00358**
mmu-miR-224	0.765	0.00375
mmu-miR-222	0.751	0.00489
mmu-let-7c	0.747	0.00523
mmu-miR-187	-0.747	0.00526
mmu-miR-1954	0.728	0.00727
mmu-miR-30c	-0.724	0.0078
mmu-miR-2146	-0.722	0.00805
mmu-miR-335-5p	-0.714	0.00911
mmu-miR-2138	0.712	0.00943
**mmu-miR-145**	**-0.707**	**0.0101**

In bold are 6 miRNAs, which are expressed in all course of infection.

### Target prediction and pathway analyses

**Fig 6:**
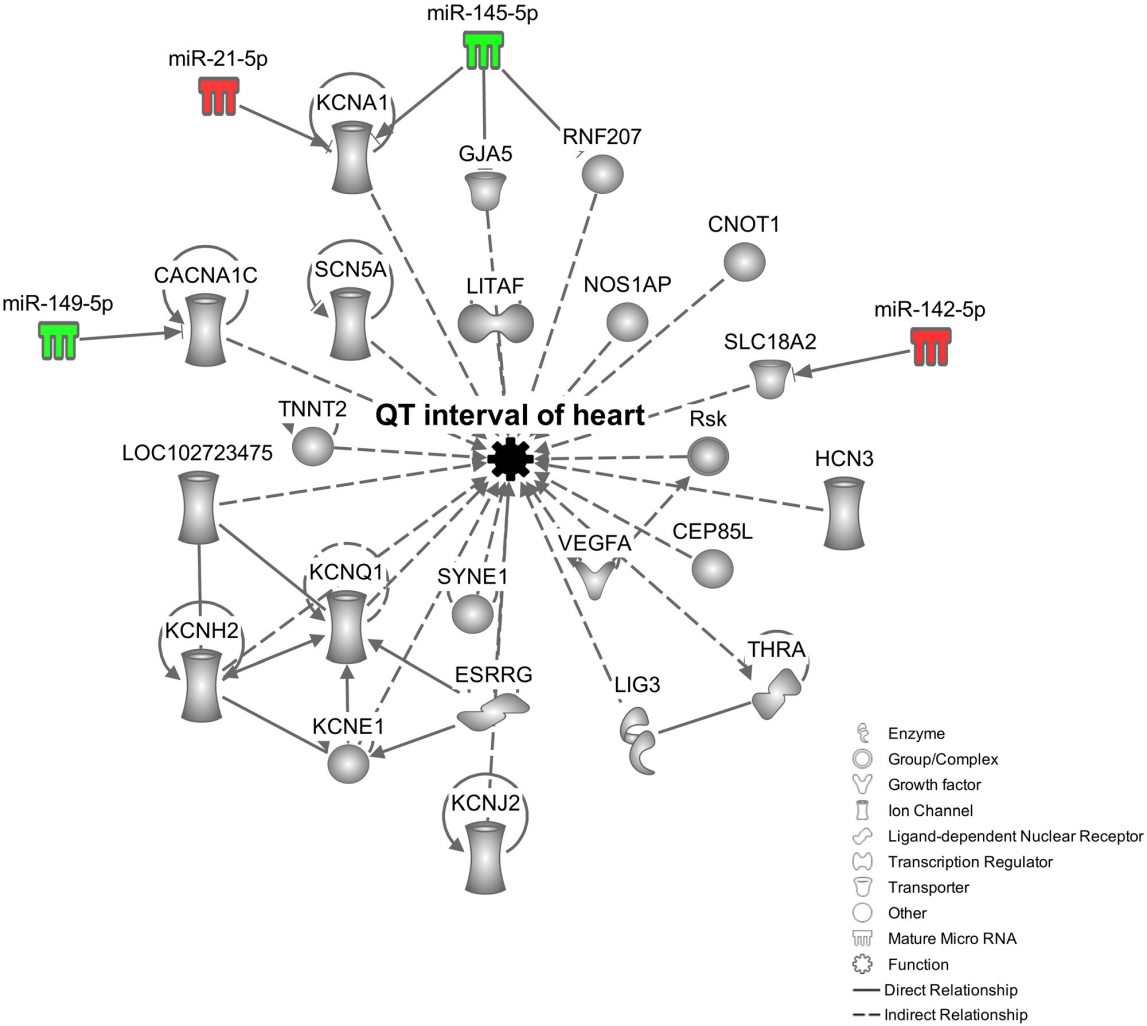
Network of miRNAs and molecules related to heart QT interval regulation. *In silico* analysis done using the IPA software (Qiagen, USA) showing a biological network built with 4 miRNAs (miR-142-5p, miR-21-5p, miR-145-5p and miR-149-5p) from the 6 (miR-146b, miR-21-5p, miR-142-3p, miR-142-5p, miR-145-5p and miR-149) uploaded for the analysis. The resulted network shows how the 4 miRNAs and the multiple molecules that are regulated directly or indirectly by them are related to the regulation of heart QT interval. The miRNAs are represented in graduation of red and green based on their fold change in expression at 45 dpi compared to 0 dpi.

Ingenuity Pathway Analysis (IPA) software was used to identify molecular networks and targets of the miRNAs miR-146b, miR-21, miR-142-3p miR-142-5p, miR-145 and miR-149, which were differentially expressed in all three time points post infection and were significantly correlated both with changes in parasitemia and QTc interval. A biological network was built in order to investigate the connection between those six miRNAs and the QTc interval (Fig 6). As observed, four out of those six miRNAs (miR-142-5p, miR-21-5p, miR-145-5p and miR-149-5p), directly or indirectly regulate several genes involved with QTc interval length. This network reveals five putative direct targets: CACNA1C (Calcium channel) target of miR-149-5p; GJA5 (Gap Junction protein, alpha 5), RNF207 (Ring finger protein 207) and KCNA1 (potassium voltage-gated channel shaker-related subfamily, member 1) targets of miR-145-5p; KCNA1 which is also a miR-21-5p target; and finally, SLC18A2 (Solute carrier family 18 member 2) target of miR-142-5p. In Fig 6 the miRNAs are represented in graduation of red and green based on their fold change in expression at 45 dpi compared to 0 dpi.

QTc interval predicted target genes show opposite fold change direction to their miRNAs

**Fig 7:**
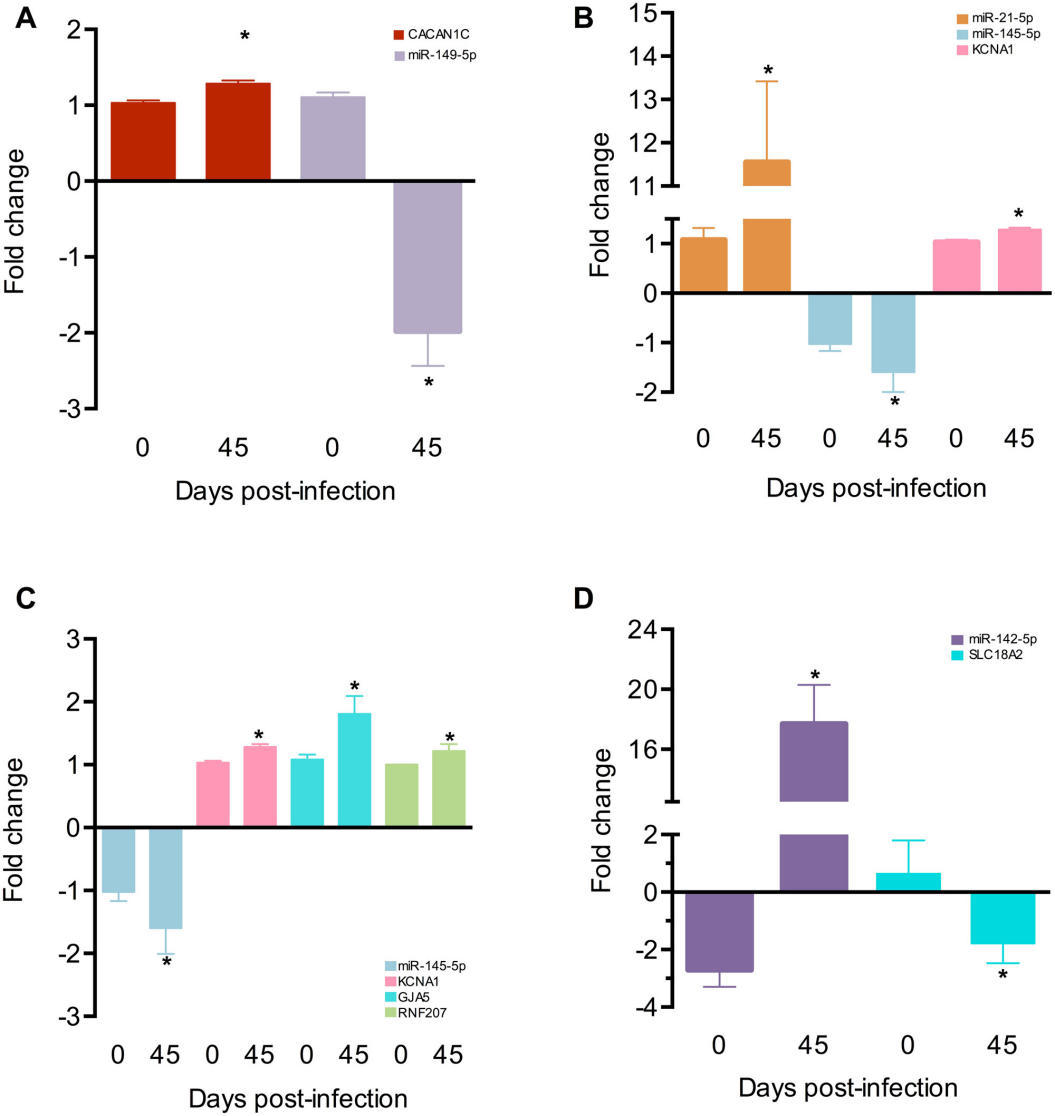
Individual mature miRNAs and their respective putative gene targets: A) miR-149-5p and CACAN1C B) miR-21-5p, miR-145-5p and KCNA1 C) miR-145-5p and KCNA1, GJA5, RNF207 and D) miR-142-5p and SLC18A2 were measured by real time RT-PCR in each time point post infection (15, 30 and 45) in four animals per group. The expression was calculated as the mean ± s.d. for each group as individual data points using the relative expression (fold change over CONT) by the 2^-ΔΔct^ method, where Ct is the threshold cycle. Groups were compared by a non-parametrical test (Mann-Whitney Rank Sum Test) with GraphPad Prism software (version 5.0.4). Results were expressed as medians and interquartile ranges. * P-values were considered significant if <0.05.

Fig 7 shows the real time RT-PCR results comparing the fold change between the miRNAs and their corresponding targets at 0 dpi and 45 dpi. A concordant expression pairing pattern between miRNAs and targets was observed—the miRNAs expression increased while their corresponding targets decreased, and vice versa. Fig 7A shows CACAN1C upregulated at 45 dpi while miR-149-5p is downregulated, both compared to their controls. In the same way, KCNA1 is upregulated while miR145-5p is downregulated, however, miR-21-5p is also upregulated (Fig 7B). This also happens with miR-145-5p and its targets KCNA1, GJA5 and RNF207 (Fig 7C). Conversely, SLC18A2 gene is downregulated while miR-142-5p is upregulated (Fig 7D). All targets and miRNAs fold change in expression were compared with their controls. S1 Fig shows these results also represented as a network.

## Discussion

Taken together, our results show that acute infection of mice with *T*. *cruzi* was able to modify the microRNA expression profile of the heart. The study of the acute phase of human Chagas disease is limited by the scarcity of samples, since most of the acute cases are not reported, because of the mild or absent symptomatology [24]. Despite all limitations, experimental models can serve as an important tool for the understanding of the early moments of the infection. As expected for the *T*. *cruzi* colombiana model of infection, our experiments showed that the mice were successfully infected with high blood and heart parasitism, myocarditis and fibrillation. The infected mice presented ECG abnormalities after 30 dpi with significantly increased repolarization parameters compared to control. Parasitemia and QTc interval are important parameters in the infection by *T*. *cruzi*. It has been found that the parasite load in the acute phase affects the prognosis in the chronic phase of Chagas disease in mouse models [7]. In addition, some clinical features of Chagas disease suggest the importance of abnormal ventricular repolarization in its pathogenesis, such as dilatation, hypertrophy, arrhythmias and sudden death [25–28] The present work is in agreement with previous reports that show that *T*. *cruzi* infection significantly alters ECG ventricular repolarization parameters, like the QTc interval [28,29]. It was reported that the QTc interval dispersion is presumed to be an important mortality risk predictor in patients with Chagas disease as well as in mice infected with certain parasite strains [25–27,29]. In our data, the relationship between QTc and parasitemia with the evolution of the disease is clear. QTc wave broadening coincides with the onset peak of parasitemia. MicroRNAs are highly conserved molecules, with analogous regulation behaviours across species [30], which allows us to extrapolate and believe that differentially expressed microRNAs in mice may play relevant roles in humans as well. In the present work, we identified a cluster of microRNAs, which the expression pattern correlated with the clinical parameters parasitemia and QTc interval in the three analysed time points. MicroRNA profiles have been recognized as impressive phenotypic signatures in heart diseases, even more effective than microarrays containing thousands of protein-coding genes [31]. The microRNA expression profile of acutely *T*. *cruzi*-infected mice was altered with an increasing number of dysregulated microRNAs as the infection progressed. In addition, this profile segregated samples according to the time of infection, both in the unsupervised clustering and in the PCA analyses, representing a reliable discriminative profile of the *T*. *cruzi* infection. Nine out of the 17 miRNAs common to the three time points presented a consistent expression pattern in the heatmap analysis. Furthermore, these miRNAs follow a similar kinetics along all the evaluated time points and have an absolute maximum fold change at 30 dpi. The same time point that the infection reaches the highest parasitemia. Although, this study focus in the acute phase of the experimental Chagas disease some miRNAs (miR-133, miR-208) were found down regulated at 45 dpi in accordance with previously reported in human heart of Chagas chronic patients [15].

Correlation analysis identified potential miRNAs related to the clinical parameters. In addition, the pathway analysis revealed putative relationship between miRNAs and their targets, and how they could influence the ECG parameters. CACNA1C is a calcium channel responsible for the L type current. This molecule is expressed in all excitable heart cells. A previous study showed this current is highly altered during experimental Chagas disease [32,33]. This gene was highly predicted as a miR-149-5p target presenting two seed sequences. KCNA1 is a potassium channel massively expressed in neurons and its expression deregulation may facilitate the occurrence of atrial fibrillation [34]. This gene was also highly predicted as target of both miRNAs miR-21-5p and miR-145-5p. These miRNAs have opposite expression direction so that KCNA1 expression remaining almost unaltered. miR-145-5p also has two other targets, GJA5 and RNF207 of which both are upregulated. GJA5 codes for the gap junction connexin-40 (Cx40), molecule responsible for electrical impulse conduction in the heart and is associated with atrial fibrillation [35]. In turn, RNF207 is one of the main potassium channels related to repolarization of the cardiac action potential and it was recently associated with the regulation of humans QTc interval [36]. Finally, SLC18A2 codes for vesicular monoamine type 2 transporter, a molecule necessary for the vesicular release of the neurotransmitters [37]. According to our network analysis, it was predicted as a miR-142-5p target. In fact, the real time RT-PCR results showed that all these miRNAs and targets follow an expression-pairing pattern, supporting the network analysis. Therefore, such altered expression of the microRNAs, may modify the expression of their target molecules, contributing to the prolongation of QTc interval during *T*. *cruzi* infection. The alterations occurring in the host microRNA profile observed here reflect the role of these molecules in the acute phase of the infection and may highlight important aspects of the pathogenesis, opening a broad range of possibilities in the study of Chagas disease

## Supporting Information

S1 Fig Network of miRNAs and molecules related to heart QT interval regulation. *In silico* analysis done using the IPA software (Ingenuity Systems, USA) showing a biological network built with four miRNAs (miR-142-5p, miR-21-5p, miR-145-5p and miR-149-5p). The resulted network shows how the four miRNAs and their putative targets have an expression pairing pattern between miRNAs and their targets—the miRNAs expression increased (in red) while their corresponding targets decreased (in green), and vice versa. The miRNAs are represented in graduation of red and green based on their fold change in expression at 45 dpi compared to 0 dpi.
(TIF)

S1 Table List of miRNAs analyzed in the study with miRBase accession number, mature miRNA sequence and chromosome location. In bold 113 miRNAs with significant differentially expression (p≤ 0,05) in at least one time point and fold change differences comparing *T*. *cruzi* infected groups (15, 30 and 45 dpi) over Control.
(DOCX)

S2 Table Correlations between microRNAs expression and parasitemia. List of 73 microRNAs with a significant correlation with parasitemia.
(DOCX)

S3 Table Correlations between microRNAs expression and QTc interval. List of 67 microRNAs with a significant correlation with QTc interval.
(DOCX)
