# Supplementary figures and images for: MicroRNA Transcriptome Profiling in Heart of Trypanosoma cruzi-Infected Mice: Parasitological and Cardiological Outcomes
Source: PLoS Negl Trop Dis. 2015 Jun 18;9(6):e0003828. doi: 10.1371/journal.pntd.0003828 (PMC4473529; doi:10.1371/journal.pntd.0003828)

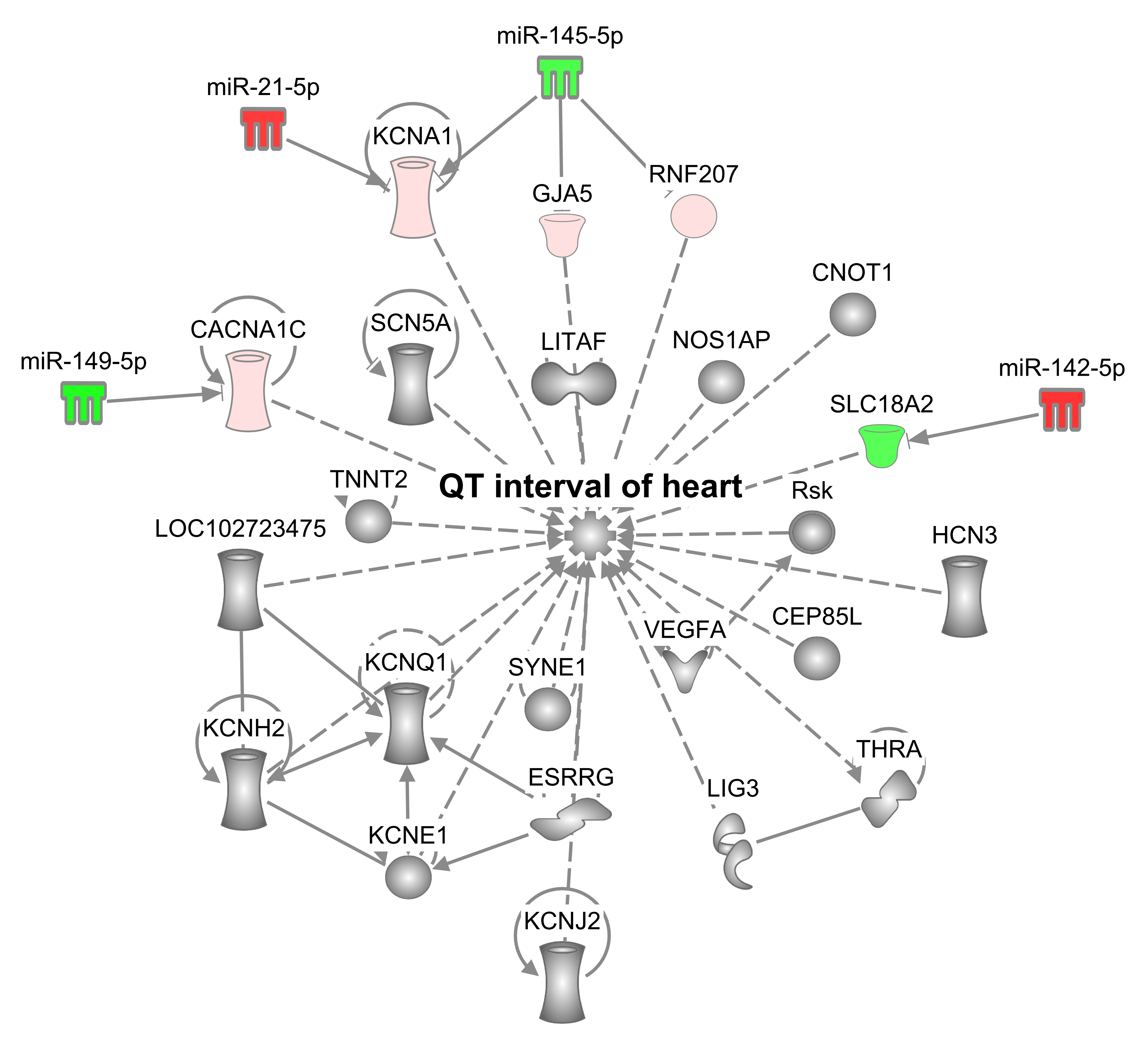

Supplement: S1 Fig — In silico analysis done using the IPA software (Ingenuity Systems, USA) showing a biological network built with four miRNAs (miR-142-5p, miR-21-5p, miR-145-5p and miR-149-5p). The resulted network shows how the four miRNAs and their putative targets have an expression pairing pattern between miRNAs and their targets—the miRNAs expression increased (in red) while their corresponding targets decreased (in green), and vice versa. The miRNAs are represented in graduation of red and green based on their fold change in expression at 45 dpi compared to 0 dpi. (TIF) [file pntd.0003828.s001.tif]
