# Supplementary material for: MicroRNA Transcriptome Profiling in Heart of Trypanosoma cruzi-Infected Mice: Parasitological and Cardiological Outcomes
Source: PLoS Negl Trop Dis. 2015 Jun 18;9(6):e0003828. doi: 10.1371/journal.pntd.0003828 (PMC4473529; doi:10.1371/journal.pntd.0003828)
Supplement: S1 Table — In bold 113 miRNAs with significant differentially expression (p≤ 0,05) in at least one time point and fold change differences comparing T. cruzi infected groups (15, 30 and 45 dpi) over Control. (DOCX) [file pntd.0003828.s002.docx]

**Supplementary Table 1**

**S1Table**.

**S1Table.** Continued
