# Supplementary material for: MicroRNA Transcriptome Profiling in Heart of Trypanosoma cruzi-Infected Mice: Parasitological and Cardiological Outcomes
Source: PLoS Negl Trop Dis. 2015 Jun 18;9(6):e0003828. doi: 10.1371/journal.pntd.0003828 (PMC4473529; doi:10.1371/journal.pntd.0003828)
Supplement: S2 Table — List of 73 microRNAs with a significant correlation with parasitemia. (DOCX) [file pntd.0003828.s003.docx]

**Supplementary Table 2**

**S2Table**

| **MicroRNA** | **R** | **p-value** |
| --- | --- | --- |
| mmu-miR-142-5p | 0.95 | 1.88.10^-8^ |
| mmu-miR-146a | 0.944 | 3.84.10^-8^ |
| mmu-miR-142-3p | 0.934 | 1.21.10^-7^ |
| mmu-miR-21 | 0.931 | 1.71.10^-7^ |
| mmu-miR-155 | 0.918 | 5.35.10^-7^ |
| rno-miR-146b | 0.903 | 1.63.10^-6^ |
| rno-miR-20b | 0.895 | 2.76.10^-6^ |
| mmu-miR-146b | 0.894 | 3.13.10^-6^ |
| mmu-miR-222 | 0.884 | 5.46.10^-6^ |
| mmu-miR-21 | 0.884 | 5.63.10^-6^ |
| mmu-miR-30e | -0.881 | 6.70.10^-6^ |
| mmu-miR-145 | -0.878 | 7.79.10^-6^ |
| mmu-miR-130b | 0.874 | 9.85.10^-6^ |
| mmu-miR-203 | 0.871 | 1.14.10^-5^ |
| mmu-miR-503 | -0.87 | 1.20.10^-5^ |
| mmu-miR-204 | -0.867 | 1.37.10^-5^ |
| mmu-miR-126-3p | -0.863 | 1.66.10^-5^ |
| mmu-miR-15b-3p | 0.861 | 1.85.10^-5^ |
| mmu-miR-322 | -0.86 | 1.89.10^-5^ |
| mmu-miR-690 | 0.86 | 1.92.10^-5^ |
| mmu-miR-342-3p | 0.857 | 2.26.10^-5^ |
| mmu-miR-126-5p | -0.853 | 2.73.10^-5^ |
| mmu-miR-139-5p | -0.847 | 3.44.10^-5^ |
| mmu-miR-26a | -0.844 | 4.02.10^-5^ |
| mmu-miR-192 | -0.842 | 4.31.10^-5^ |
| mmu-miR-195 | -0.841 | 4.49.10^-5^ |
| mmu-miR-26b | -0.839 | 4.80.10^-5^ |

**S2Table - Continued**

| **MicroRNA** | **R** | **p-value** |
| --- | --- | --- |
| mmu-miR-875-5p | 0.827 | 7.71.10^-5^ |
| mmu-miR-149-5p | -0.826 | 8.10.10^-5^ |
| mmu-miR-143 | -0.815 | 0.00012 |
| rno-miR-1 | -0.815 | 0.00012 |
| mmu-miR-30a-3p | -0.814 | 0.000123 |
| mmu-miR-210 | 0.81 | 0.000142 |
| mmu-miR-182 | 0.805 | 0.00017 |
| mmu-miR-1971 | 0.803 | 0.000178 |
| mmu-miR-499 | -0.794 | 0.00024 |
| mmu-miR-328 | -0.788 | 0.000288 |
| mmu-miR-320 | -0.784 | 0.000323 |
| mmu-miR-20b | 0.77 | 0.000479 |
| mmu-miR-345-5p | -0.768 | 0.000511 |
| mmu-miR-30b | -0.765 | 0.000549 |
| mmu-miR-30b | 0.761 | 0.000619 |
| mmu-miR-30e-3p | -0.757 | 0.000686 |
| mmu-miR-805 | -0.745 | 0.000925 |
| mmu-miR-1839-5p | 0.735 | 0.00119 |
| mmu-miR-133b | -0.734 | 0.00121 |
| mmu-miR-652 | 0.732 | 0.00127 |
| mmu-miR-409-3p | 0.718 | 0.00175 |
| mmu-miR-130b | 0.714 | 0.0019 |
| mmu-miR-342-5p | 0.712 | 0.00196 |
| mmu-miR-208 | -0.711 | 0.00204 |
| rno-miR-664 | -0.71 | 0.00206 |
| mmu-miR-135a | -0.71 | 0.00207 |
| mmu-miR-449a | 0.703 | 0.00239 |

**S2Table - Continued**

| **MicroRNA** | **R** | **p-value** |
| --- | --- | --- |
| mmu-miR-185 | -0.697 | 0.00271 |
| mmu-miR-187 | -0.694 | 0.00285 |
| mmu-miR-362-3p | 0.693 | 0.00293 |
| mmu-miR-1 | -0.692 | 0.00298 |
| mmu-miR-218 | -0.68 | 0.00374 |
| mmu-miR-30a | -0.68 | 0.00378 |
| mmu-miR-335-3p | -0.677 | 0.00399 |
| mmu-miR-137 | -0.669 | 0.0046 |
| mmu-miR-467b | 0.668 | 0.00465 |
| mmu-miR-744 | -0.665 | 0.00495 |
| mmu-miR-494 | 0.665 | 0.00498 |
| mmu-miR-133a | -0.66 | 0.0054 |
| mmu-miR-27a | 0.657 | 0.00568 |
| mmu-miR-322 | -0.653 | 0.00607 |
| mmu-miR-494 | -0.646 | 0.00682 |
| mmu-miR-331-3p | -0.641 | 0.0075 |
| mmu-miR-152 | -0.64 | 0.00763 |
| mmu-miR-125b-5p | -0.636 | 0.00814 |
| mmu-miR-24 | -0.634 | 0.00842 |
| mmu-miR-2138 | 0.631 | 0.0087 |
| mmu-miR-101a | -0.629 | 0.00901 |
