# Supplementary material for: MicroRNA Transcriptome Profiling in Heart of Trypanosoma cruzi-Infected Mice: Parasitological and Cardiological Outcomes
Source: PLoS Negl Trop Dis. 2015 Jun 18;9(6):e0003828. doi: 10.1371/journal.pntd.0003828 (PMC4473529; doi:10.1371/journal.pntd.0003828)
Supplement: S3 Table — List of 67 microRNAs with a significant correlation with QTc interval. (DOCX) [file pntd.0003828.s004.docx]

**Supplementary Table 3**

**S3 Table**

| **MicroRNA** | **R** | **p-value** |
| --- | --- | --- |
| mmu-miR-149-5p | -0.926 | 2.76.10^-7^ |
| mmu-miR-320 | -0.873 | 1.01.10^-5^ |
| mmu-miR-30a-3p | -0.871 | 1.11.10^-5^ |
| mmu-miR-21 | 0.868 | 1.32.10^-5^ |
| mmu-miR-142-5p | 0.855 | 2.47.10^-5^ |
| mmu-miR-30e-3p | -0.85 | 3.00.10^-5^ |
| mmu-miR-139-5p | -0.849 | 3.19.10^-5^ |
| mmu-miR-503 | -0.849 | 3.20.10^-5^ |
| mmu-miR-142-3p | 0.84 | 4.71.10^-5^ |
| mmu-miR-126-3p | -0.82 | 0.000101 |
| mmu-miR-322 | -0.818 | 0.000109 |
| mmu-miR-30b | 0.807 | 0.000156 |
| mmu-miR-335-5p | -0.807 | 0.000158 |
| mmu-miR-146b | 0.803 | 0.000181 |
| mmu-miR-30b | -0.802 | 0.000189 |
| mmu-miR-30c | -0.8 | 0.000199 |
| rno-miR-146b | 0.797 | 0.00022 |
| mmu-miR-147 | 0.794 | 0.000237 |
| mmu-miR-187 | -0.79 | 0.000272 |
| mmu-miR-155 | 0.784 | 0.000328 |
| mmu-miR-204 | -0.782 | 0.000345 |
| mmu-miR-145 | -0.781 | 0.000354 |
| mmu-miR-15b | 0.772 | 0.000457 |
| rno-miR-345-3p | -0.77 | 0.000482 |
| mmu-miR-652 | 0.768 | 0.000506 |
| mmu-miR-345-5p | -0.766 | 0.000536 |
| mmu-miR-192 | -0.759 | 0.000648 |

**S3 Table** – Continued

| **MicroRNA** | **R** | **p-value** |
| --- | --- | --- |
| mmu-miR-200a | 0.753 | 0.000765 |
| mmu-miR-34b-3p | 0.752 | 0.000775 |
| mmu-miR-30e | -0.752 | 0.000783 |
| mmu-miR-186 | -0.751 | 0.000797 |
| mmu-miR-328 | -0.738 | 0.00111 |
| mmu-miR-133a | -0.736 | 0.00116 |
| mmu-miR-342-3p | 0.733 | 0.00125 |
| mmu-miR-342-5p | 0.731 | 0.00129 |
| mmu-miR-222 | 0.727 | 0.00141 |
| mmu-miR-9 | -0.725 | 0.00148 |
| mmu-miR-805 | -0.722 | 0.0016 |
| mmu-miR-378 | -0.721 | 0.00163 |
| mmu-miR-210 | 0.712 | 0.00198 |
| mmu-miR-146a | 0.719 | 0.00168 |
| mmu-miR-130b | 0.712 | 0.00199 |
| mmu-miR-195 | -0.698 | 0.00266 |
| mmu-miR-499 | -0.692 | 0.00297 |
| rno-miR-1 | -0.688 | 0.00321 |
| mmu-miR-143 | -0.684 | 0.00346 |
| mmu-miR-494 | 0.679 | 0.00382 |
| mmu-miR-143 | -0.672 | 0.00437 |
| mmu-miR-34c | 0.671 | 0.0044 |
| mmu-miR-331-3p | -0.671 | 0.00443 |
| rno-miR-7 | -0.67 | 0.0045 |
| mmu-miR-15b | 0.668 | 0.00472 |
| mmu-miR-26b | -0.667 | 0.00475 |
| mmu-miR-337 | 0.665 | 0.00498 |
| mmu-miR-2146 | -0.662 | 0.00521 |

**S3 Table** – Continued

| **MicroRNA** | **R** | **p-value** |
| --- | --- | --- |
| rno-miR-664 | -0.661 | 0.00527 |
| mmu-miR-322 | -0.659 | 0.00546 |
| mmu-miR-466 | 0.654 | 0.00603 |
| mmu-miR-133b | -0.65 | 0.00643 |
| mmu-miR-130a | 0.649 | 0.00656 |
| rno-miR-204 | -0.645 | 0.00699 |
| mmu-miR-215 | 0.644 | 0.00713 |
| mmu-miR-376a | 0.643 | 0.00726 |
| mmu-miR-213 | -0.638 | 0.00784 |
| mmu-miR-7b | 0.634 | 0.00841 |
| mmu-miR-1971 | 0.631 | 0.00881 |
| mmu-miR-2138 | 0.631 | 0.00881 |
| mmu-miR-362-3p | 0.628 | 0.00919 |
| rno-miR-20b | 0.625 | 0.00966 |
| mmu-miR-467a | 0.625 | 0.00968 |
| mmu-miR-690 | 0.624 | 0.00972 |
| mmu-miR-27a | 0.624 | 0.00979 |
